# Comparison of barbed suture and conventional suture in laparoendoscopic single-site myomectomy

**DOI:** 10.3389/fonc.2025.1541002

**Published:** 2025-05-01

**Authors:** Weimin Xie, Shuiping Tang, Zhangyi Wang, Xiaohang Liu, Li Wang, Rong Tang, Chaoqun Tang, Chunyan Xie

**Affiliations:** ^1^ Department of Gynecology, Affiliated Hengyang Hospital of Hunan Normal University and Hengyang Central Hospital, Hengyang, Hunan, China; ^2^ Department of General Surgery, Affiliated Hengyang Hospital of Hunan Normal University and Hengyang Central Hospital, Hengyang, Hunan, China; ^3^ Nursing Department, Affiliated Hengyang Hospital of Hunan Normal University and Hengyang Central Hospital, Hengyang, Hunan, China; ^4^ Department of Gynecology, Hainan General Hospital, Hainan Affiliated Hospital of Hainan Medical University, Haikou, Hainan, China; ^5^ Department of Gynecology, Tongren People’s Hospital, Tongren, Guizhou, China; ^6^ Department of Gynecology, Affiliated Nanhua Hospital, Hengyang Medical School, University of South China, Hengyang, Hunan, China

**Keywords:** laparoendoscopic single-site myomectomy, barbed suture, conventional suture, uterine myomas, feasibility

## Abstract

**Objective:**

To compare surgical outcomes of laparoendoscopic single-site myomectomy (LESS-M) for uterine myomas using barbed suture versus conventional suture.

**Methods:**

Data were collected from all women with uterine myomas who underwent LESS-M at three institutions. Patients were managed by LESS-M with either barbed suture or conventional suture.

**Results:**

Operative time was significantly lower in the barbed suture group in comparison with the conventional suture group (65.4 ± 10.7 min vs. 78.02 ± 14.2 min, P = 0.000). Similarly, the amount of blood loss was lower in the barbed suture group than in the conventional suture group (158.1 ± 85.2 mL versus 209.6 ± 85.9, P = 0.000). Accordingly, the change in hemoglobin levels in the barbed suture group was lower than in the conventional suture group (16.6 ± 5.9 g/L versus 21.0 ± 4.8, P = 0.000). Conversely, there were no statistically significant differences for blood transfusion, the postoperative pain VAS score assessed at 24 hours, length of hospital stay, conversion, and perioperative complication rates between the two groups (*P* > 0.05 for all).

**Conclusion:**

The use of barbed suture may reduce operative time, blood loss, and hemoglobin change during LESS-M, which may be an optimal and efficient alternative to conventional suture.

## Introduction

Uterine myomas, also known as leiomyomas or fibroids, are the most common gynecologic benign neoplasms in women of reproductive age (1). Their management mainly depends on symptoms, such as menorrhagia, pelvic pressure/pain, or infertility. Myomectomy is the typical surgical management option for various women who suffer from myomas and have a strong desire for preserving their fertility. Laparoscopic myomectomy is generally considered to be a suitable alternative to standard laparotomic myomectomy for managing uterine myomas. Indeed, when compared with laparotomic myomectomy, laparoscopic myomectomy is associated with shorter hospitalization, lower perioperative complications, and lower pain scores, and it yields better cosmetic results (2, 3). However, laparoscopic myomectomy is a challenging procedure, especially with regards to the repair of uterine wall defects and reduce bleeding during myomectomy.

Transumbilical laparoendoscopic single-site surgery (LESS) is a minimally invasive strategy that has been widely used to treat benign gynecologic diseases including ectopic pregnancies, fibroids, ovarian cysts, and ovarian endometriomas. The main advantages of LESS are that only a single surgical approach is required, and although the scar is concealed in the umbilicus, the incision is sufficiently large to conveniently retrieve the specimen (4–6). LESS has been reported for many years, however, this procedure did not initially gain popularity because of the technical challenges. LESS has resurged again due to the recent technological advances in endoscopic instrumentation and optics (7, 8). However, LESS myomectomy (LESS-M) has not been widely performed due to its technical difficulties. Suturing and knotting are considered by many surgeons to be the most challenging laparoscopic skills and time-consuming components of LESS, which require extensive training (9, 10). In addition, the absence of a surgical operating triangle, the limited range of motion between instruments, and the lack of assistance in laparoscopy leads to mutual interference among the instruments and increases the difficulty of surgery.

Barbed suture is a new technology that has the potential to greatly facilitate laparoscopic suturing and knotting. One of these novel sutures, the V-Loc (Covidien, Mansfield, MA) consists of a unidirectional barbed absorbable thread armed with a loop at one end and a surgical needle at the opposite end to secure the suture. These barbs and loop ends simplify continuous suturing by eliminating the need to tie a surgical knot. Barbed suture has been widely used with good results in a number of multi-port laparoscopic gynecologic surgeries, such as myomectomy (11) and hysterectomy (12).

In the present study, we analyzed a large cohort of women of reproductive age diagnosed with uterine myomas, therefore potentially eligible for LESS-M. The aim of this study was to compare clinical outcomes in terms of the feasibility, safety, and efficacy of LESS-M for uterine myomas using a unidirectional knotless barbed suture versus conventional smooth suture.

## Materials and methods

A retrospective study, data were obtained for all women with uterine myomas who underwent LESS-M between January 1, 2019, and January 31, 2024, at the Department of Gynecology of Hengyang Central Hospital (Hengyang, China), the Department of Gynecology of Hainan General Hospital (Haikou, China), or the Department of Gynecology of Tongren People’ s Hospital (Tongren, China). The study was conducted with the approval of the institutional ethics committee of the hospitals. All LESS-M procedures were conducted by surgeons experienced in over 100 laparoscopic myomectomies. All women provided informed consent for the surgical procedure after receiving thorough counseling about their therapeutic options. The women were informed that laparotomy would be undertaken if difficulties were encountered with the laparoscopic approach.

The inclusion criteria were: (1) age between 18 and 50 years; (2) women with myomas causing symptoms such as menorrhagia, pelvic pressure/pain, or infertility; (3) women who wish to preserve fertility; (4) women who had myomas no larger than 10 cm and no more than three intramural myomas; (5) appropriate medical status for laparoscopic surgery (American Society of Anesthesiologists Physical Status classification 1 or 2). The exclusion criteria were: (1) women with a dominant pedunculated subserosal or submucosal type myoma; (2) previous history of myomectomy; (3) additional diseases requiring surgical treatment (such as endometriosis, tubal surgery, severe adhesiolysis); (4) women with any suggestion or history of malignant uterine or adnexal diseases. Pregnant patients were also ineligible for this surgery. No patient included in the study underwent medical treatment for ovarian suppression before surgery. Based on the type of suture used during surgery, eligible patients were divided into the barbed suture group or the conventional suture group. Patients with insufficient clinical data or who were lost to follow-up immediately after surgery were excluded.

The patient was placed approximately in a 30-degree Trendelenburg position under general anesthesia. After making a 20–30 mm vertical skin incision in the umbilicus, a commercially available, 4-channel, single-port system was inserted. All participating surgeons had comparable surgical skills and a preference for LESS surgery. After the pelvic organs were explored, a dilute vasopressin was injected into the serosal and/or overlying myometrium, and just around the myoma, to reduce blood loss. An incision was made through the uterine wall and the pseudocapsule of the myoma by use of a unipolar hook scissor. After identifying the cleavage plane, the myoma was enucleated by means of adequate traction with a laparoscopic myoma drill or grasping forcep. Coagulation of significant bleeding vessels was performed by bipolar forceps. After enucleation of myomas, the uterine walls were sutured in two layers with a continuous suture. In the barbed suture group, a 2–0 polyglyconate monofilament absorbable barbed suture (V-Loc 180; Covidien, Mansfield, MA) was used for suturing. In the conventional suture group, a 2–0 polyglactin 910 suture (Vicryl; Ethicon, Somerville, NJ) was used for suturing. After repair of the myometrium was completed, the myomas were extracted through the umbilical incision by cutting them with a knife in the specimen retrieval bag or the surgical glove.

Data for all women meeting the inclusion criteria were collected from the medical records, including patient characteristics, outcome measurements, and intraoperative and postoperative complications. Operative time was defined as the time from the induction of pneumoperitoneum to desufflation. Blood loss was calculated as the difference between the total amount of suction and irrigation plus the difference between the total gauze weight before and after surgery. Conversion was defined as either the placement of additional port(s) or conversion to abdominal ovarian cystectomy, while conversion performed due to the requirement for additional surgery was excluded. Patients were discharged from the hospital when they were mobile with well-controlled pain, tolerated an oral diet, and resumed normal bowel and urinary functions.

Statistical analyses were performed using SPSS version 18. Continuous data were tested for normality using the Shapiro-Wilk test. Normally distributed variables were expressed as mean ± standard deviation (SD) and compared using the Student t test. Non-normally distributed continuous data were expressed as median and interquartile range (IQR) and compared using the Mann-Whitney U test. Categorical variables were expressed as counts and percentages and compared using the χ² test or Fisher’s exact test as appropriate. Given the limited number of primary outcome comparisons and clear hypotheses, no adjustment for multiple comparisons was performed. All tests were two-sided, and P < 0.05 was considered statistically significant. Baseline characteristics were balanced between the groups, so no additional multiple regression analysis was performed.

## Results

During the study, 210 women with symptomatic myomas fulfilled the inclusion and exclusion criteria. Of these, 100 patients underwent barbed suture in LESS-M, and 110 patients underwent conventional suture in LESS-M. Patient baseline characteristics are shown in **Table 1**. There were no statistically significant differences among patients with respect to age, body mass index, parity, abdominal surgical history, and preoperative hemoglobin (*P* > 0.05 for all). The mean diameter of the largest myoma was 6.9 ± 1.4 cm for the barbed suture group and 6.7 ± 1.2 cm for the conventional suture group; the difference between the 2 groups was not statistically significant (*P* > 0.05). The average number of uterine myomas was 1.6 ± 0.7 for the barbed suture group and 1.7 ± 0.6 for the conventional suture group; the difference between the 2 groups was not statistically significant (*P* > 0.05). The main indication for myomectomy, location of largest myoma, and presence of adhesions also did not show statistical significances between the two groups (*P* > 0.05 for all).

**Table 1:** Patients baseline characteristics.

Characteristics	Barbed suture (n = 100)	Conventional suture (n =110)	*P* value
Age (years)	33.4 ± 5.7	32.1 ± 5.8	0.103
Body mass index (kg/m^2^)	25.0 ± 2.3	24.8 ± 2.4	0.487
Parity			
0	35 (35.0%)	38 (34.5%)	
≥ 1	65 (65.%)	72 (65.5%)	0.945
Abdominal surgical history			
No	77 (77.0%)	84 (76.4%)	
Yes	23 (23.0%)	26 (23.6%)	0.913
Main indication for myomectomy			0.998
Menorrhagia	53 (53.0%)	58 (52.7%)	
Pelvic pain or pressure	27 (27.0%)	29 (26.4%)	
Rapid growing or infertility	20 (20.0%)	23 (20.9%)	
Number of uterine myomas	1.6 ± 0.7	1.7 ± 0.6	0.243
Diameter of largest myoma (cm)	6.9 ± 1.4	6.7 ± 1.2	0.485
Location of largest myoma			0.622
Anterior	47 (47.0%)	42 (38.2%)	
Posterior	43 (43.0%)	49 (44.5%)	
Lateral or fundal	10 (10.0%)	19 (17.3%)	
Presence of adhesions			
No	79 (79.0%)	86 (78.2%)	
Yes	21 (21%)	24 (21.8%)	0.885

Data are expressed as mean ± SD, or No. (%), as appropriate.

The surgical outcomes of each group are shown in **Table 2**. Operative time was significantly lower in the barbed suture group in comparison with the conventional suture group (65.4 ± 10.7 min vs. 78.02 ± 14.2 min, *P* = 0.000). Similarly, the amount of blood loss was lower in the barbed suture group than in the conventional suture group (158.1 ± 85.2 mL versus 209.6 ± 85.9, *P* = 0.000). Accordingly, the change in hemoglobin levels in the barbed suture group was lower than in the conventional suture group (16.6 ± 5.9 g/L versus 21.0 ± 4.8, *P* = 0.000). Blood transfusion was necessary in three patients in the barbed suture group and five patients in the conventional suture group, respectively; the difference between the 2 groups was not statistically significant (*P* =0.823). The postoperative pain VAS score assessed at 24 hours, length of hospital stay, conversion, and perioperative complication rates also did not show statistical significances between the two groups (*P* > 0.05 for all). Conversion to multi-port laparoscopic myomectomy was reported for similar proportions of patients who underwent LESS-M using barbed suture and LESS-M with conventional suture. Conversion to multi-port laparoscopic myomectomy was needed in two patients of the barbed suture group and three patients of the conventional suture group, in order to promptly control severe uterine hemorrhage. No conversion to laparotomy occurred in both groups. There were no intraoperative complications such as organ or vessel injuries in either group. Postoperative fever was reported for similar proportions of patients who underwent LESS-M using barbed suture and LESS-M with conventional suture. Similar proportions of patients in the two groups developed a port site hematoma that resolved spontaneously without second surgery.

**Table 2:** Comparison of surgical outcomes between barbed suture and conventional suture.

Characteristics	Barbed suture (n = 100)	Conventional suture (n =110)	*P* value
Operating time, min	65.4 ± 10.7	78.02 ± 14.2	0.000
Blood loss, mL	158.1 ± 85.2	209.6 ± 85.9	0.000
Hemoglobin change (g/L)	16.6 ± 5.9	21.0 ± 4.8	0.000
Blood transfusion	3 (3.0%)	5 (4.5%)	0.823
Postoperative pain (VAS after 24 h)	3.1 ± 1.1	3.3 ± 1.3	0.297
Length of hospital stay, days	3.8 ± 0.8	3.9 ± 0.9	0.471
Conversion	2 (2.0%)	3 (2.7%)	1.000
Conversion to multi-port one^a^	2 (2.0%)	3 (2.7%)	
Conversion to laparotomy	0	0	
Perioperative complication rates, total	6 (6.0%)	8 (7.3%)	0.712
Intraoperative complications	0	0	
Bowel injury	0	0	
Postoperative complications	6 (6.0%)	8 (7.3%)	0.712
Postoperative fever	4 (4.0%)	5 (4.5%)	1.000
Wound infection	2 (2.0%)	3 (2.7%)	1.000

Data are expressed as mean ± SD, or No. (%), as appropriate.

a Indicates that the use of additional port or ports was needed.

## Discussion

The data presented in this study reveal that the unidirectional knotless barbed suture facilitates suturing of the uterine wall defect after removal of myomas during LESS-M. When compared with conventional suture, barbed suture significantly reduced the operative time, the amount of blood loss, and the change in hemoglobin levels during LESS-M.

Since the 2010s, LESS has been performed in gynecologic surgical fields, and the procedures have included salpingectomy, ovarian cystectomy, hysterectomy, and myomectomy (13–16). In recent years, clinical studies on the use of LESS-M to remove myomas have been carried out abroad (17–19), and LESS-M is considered safe and reliable compared to multi-port laparoscopic myomectomy.

The suture of the uterine wall defect is widely considered to be the most difficult and time-consuming task performed during laparoscopic myomectomy. The main reason why laparoscopic suturing is so difficult is that multiple sutures must be tied in a confined cavity with limited visibility. These limitations are even more pronounced in LESS-M due to several difficulties. First, LESS loses the surgical operating triangle offered with multi-port laparoscopic surgery. LESS enters the abdominal cavity using a single port with multi-channel, in which the surgical instruments and the camera inserted into are almost in the same plane area. Second, the limited range of motion between instruments leads to mutual interference among the instruments. Third, because of the frequent clashing between instruments and the camera, the camera cannot always provide an accurate surgical field and it is difficult to manipulate the surgical instruments smoothly. Fourth, LESS is technically difficult compared to multi-port laparoscopic surgery and has a steep learning curve.

Barbed sutures appear to be a suitable solution if a higher speed for the suture step is an issue, as well as blood loss and hemostasis barbed sutures, which allow consistent tension control over the suture line and avoid the need for knots, were first used in gynecologic surgery by Greenberg and Einarsson in 2008 (8). Barbed sutures have already proven to be a safe and effective alternative to conventional suture, with the additional benefits of reducing suturing and operative time during laparoscopic myomectomy, making suturing less difficult, and diminishing intraoperative blood loss (8, 20–22). Although the surgical advantages of barbed sutures during laparoscopic myomectomy are by now well established, the feasibility, safety, and efficacy of barbed sutures during LESS-M are not clear as yet. Our study showed that a significantly lower operation time, the amount of blood loss, and the change in hemoglobin levels during were observed with the barbed suture during LESS-M than with the conventional suture. There was no difference in the blood transfusion, postoperative pain VAS score, length of hospital stay, conversion, and perioperative complication rates duration of surgery between the barbed and conventional suture groups. In agreement with the findings of the studies concerned on laparoscopic myomectomy (8, 19–22), our data support the hypothesis that barbed sutures in LESS-M are as safe as, and an easier alternative to conventional sutures. Further randomized studies involving large populations are needed to evaluate the safety and efficacy of barbed sutures in LESS-M.

This study had some limitations. First, the study had a non-randomized comparative design, and potential biases are likely to be greater for non-randomized studies than for randomized controlled trials. Second, we did not measure the time required for suturing the uterine wall defect. Third, long-term follow-up data were not available to provide more information about results of LESS-M. We have started a 24-month follow-up study to investigate long-term outcomes.

In conclusion, barbed sutures can reduce operative time, blood loss, and hemoglobin change during LESS-M. On the basis of our results, barbed sutures could be an optimal and efficient alternative to conventional sutures to assist gynecological surgeons in performing LESS-M. To overcome the limitations of its retrospective design, more well designed, randomized controlled trials are necessary to dispel the remaining doubts and establish the safety and efficacy of barbed sutures in LESS-M.

## Data Availability

The raw data supporting the conclusions of this article will be made available by the authors, without undue reservation.
